# Delving into the complexities of the interplay between acute kidney injury and diabetic kidney disease: A focus on glycemic control and outcomes

**DOI:** 10.1590/2175-8239-JBN-2024-0074en

**Published:** 2024-12-16

**Authors:** Érika Bevilaqua Rangel

**Affiliations:** 1 Hospital Israelita Albert Einstein, Instituto Israelita de Ensino e Pesquisa, São Paulo, SP, Brazil.; 2 Universidade Federal de São Paulo, Departamento de Medicina, Divisão de Nefrologia, São Paulo, SP, Brazil.

**Keywords:** Acute Kidney Injury, Diabetes Mellitus, Diabetic Kidney Disease, Glycemic Control

## Abstract

Patients with diabetic kidney disease (DKD) face an elevated risk of experiencing acute kidney injury (AKI), exacerbating the progression of DKD. This article offers a comprehensive review of the literature and knowledge of the primary pathophysiologic mechanisms underlying kidney damage, as well as the biological implications of maladaptive kidney repair in the context of DKD complicated by AKI. Additionally, we examine in detail the findings of clinical trials evaluating the efficacy and safety of intensive insulin treatment for hyperglycemic patients in intensive care units, alongside the potential risks of hypoglycemia and mortality. Furthermore, through critical analysis of clinical trial results, opportunities for personalized safety-based approaches to mitigate side effects are identified. It is imperative to conduct randomized-controlled studies to assess the impact of intensive insulin treatment on diabetic patients with DKD, and to validate AKI biomarkers in this patient population. Such studies will help to tailor treatment strategies to improve patient outcomes and preserve kidney function.

## Introduction

### Epidemiology

Diabetes mellitus (DM) is a significant risk factor for acute kidney injury (AKI) and shows a significantly higher prevalence (ranging from 19.6 to 49%) compared to non-diabetic counterparts (ranging from 9.3% to 33%) in intensive care units (ICUs)^1^. Few studies have investigated AKI in this patient group. Table 1 provides a summary of the relevant studies^2,3,4,5,6.7,8,9,10^.

**Table 1:** Acute kidney injury and outcomes in diabetic patients

Study	Design	Sample size (n)	Objective	Results
Mehta et al.^2^	Retrospective analysis	449,524	Prevalence of DM in patient with AKI after cardiac surgery	DM prevalence is higher in AKI *vs* non-AKI patients (49% *vs* 33%, p < 0.0001)
Mittalhenkle et al.^3^	Prospective case-control analysis	5,731	AKI incidence in the elderly population	DM associated with incident AKI
Oliveira et al.^4^	Prospective analysis	980	Prevalence of DM in aminoglycoside-induced AKI	DM prevalence is higher in AKI *vs* non-AKI (19.6% *vs* 9.3%, p = 0.007)
Kheterpal et al.^5^	Retrospective, data-based analysis (American College of Surgeons National Surgical Quality Improvement Program)	75,952	AKI incidence after general surgery	Identification of DM as independent preoperative risk factor
Girman et al.^6^	Retrospective, data-based analysis (General Practice Research Database)	119,966 T2DM and 1,794,516 nondiabetic individuals	AKI in DM *vs* nondiabetic	Higher yearly AKI incidence in DM: 198 *vs* 27/100,000 subjects (crude HR = 8.0, 95% CI, 7.4–8.7)
Orban et al.^7^	Retrospective analysis	94	AKI on ICU	High blood glucose associated with increased occurrence of AKI on ICU admission
Venot et al.^8^	Prospective case-control analysis	318 diabetic and 746 nondiabetic individuals	AKI incidence in severe sepsis/septic shock	AKI incidence is not different. Higher serum creatinine and dialysis frequency in diabetic patients
Kim et al.^9^	Case-control matched analysis	884	Effect of DM on AKI after minimally invasive partial nephrectomy	Higher incidence of postoperative AKI in DM *vs* non-DM (30.7% *vs* 14.9%, p < 0.001)
Hapca et al.^10^	Retrospective analysis	9,417 T2DM and 7,283 nondiabetic individuals		Risk of AKI in DM was 5 times higher than that of controls (48.6% vs 17.2%; 121.5 *vs* 24.6 per 1000-person-years)

Importantly, the majority of individuals with DM exhibit at least one concurrent chronic disease, and up to 40% have at least three or more, including diabetic kidney disease (DKD), hypertension, heart failure, peripheral vascular disease, obesity, among others^11^. Hence, these factors contribute to a high risk of AKI.

The main causes of AKI in diabetic patients are sepsis (61%), cardiorenal syndrome (11%), prerenal syndrome (9.9%), drugs (9%), obstruction (6.6%), and contrast use (4%)^12^.

Drug-induced AKI is a significant challenge in clinical practice. Drug-associated nephrotoxicity accounts for 18–27% of all AKI cases in American hospitals^13^. In the Drug-Induced Renal Injury Consortium (DIRECT) study, the most common nephrotoxic drugs causing AKI were identified as vancomycin (48.7%), nonsteroidal anti-inflammatory drugs (NSAIDS; 18.2%), and piperacillin/tazobactam (17.8%)^14^. Besides hyperglycemia, a multivariable model identified age, vascular capacity, infections, pyuria, serum creatinine trends, and contrast media as significant predictors of drug-induced AKI, demonstrating good performance with a AUC-ROC (Area Under the Receiver Operating Characteristic Curve) of 0.86.

The pathophysiological mechanisms of drug-induced AKI vary according to the drug used^13^. Angiotensin-converting enzyme inhibitors (ACE inhibitors) and angiotensin receptor blockers (ARBs) are major contributors, causing 35% of AKI cases, primarily due to their widespread use in diabetic patients. The risk is further elevated in patients with congestive heart failure, volume depletion, those taking diuretics or NSAIDs, and those with bilateral renal artery stenosis. Aminoglycosides (such as gentamicin) and NSAIDs account for 16% of cases, followed by statins (10%), rifampicin (6%), and ifosfamide (3%). NSAIDs decrease glomerular filtration rate and renal blood flow by suppressing prostaglandin production.

Diuretic-associated AKI is characterized by tubular epithelial cell vacuolation and is more often observed in patients with higher rates of comorbidities (DM, cardiovascular disease, chronic kidney disease, and hypertension) compared to those with non-diuretic AKI^15^. Within the diuretic-induced AKI group, 27.5% of cases were attributed to diuretics alone, while 29.8% resulted from the combination of diuretics with other drugs. A retrospective study revealed that diabetic patients using hydrochlorothiazide (HCT) frequently experienced significant renal events, with an eGFR decline of over 30% in about 20% of individuals^16^.

Importantly, hypokalemia induced by diuretics may also worsen glycemic control or promote hyperglycemia, as hypokalemia impairs potassium-dependent insulin release in response to glucose overload^17^.

The evolution of diabetic patients who develop AKI indicates that about 10% experience a decline in the estimated glomerular filtration rate (eGFR), and 47% experience a decline of more than 50% in GFR, particularly if the cause of AKI was sepsis^12^.

The risk of AKI is directly proportional to the GFR (in mL/min/1.73 m^2^): 40–50 (adjusted odds ratio [aOR] 1.95), 30–44 (aOR 6.54), 15–29 (aOR 28.50), and <15 (aOR 40.07)^18^. If other comorbidities are present, such as heart failure and systemic arterial hypertension, the risk of AKI increases (OR = 3.37 and 1.94, respectively)^19^.

Of importance, the rates of AKI in diabetic patients with chronic kidney disease (CKD) are twice as high as in the non-diabetic group. After the onset of CKD, AKI rates were 384.8 per 1,000 person-years for diabetic patients compared to 180.0 per 1,000 person-years for non-diabetics^10^. Before the onset of CKD, the rates were 109.3 per 1,000 person-years for diabetic patients *vs* 47.4 per 1,000 person-years for non-diabetics.

Hence, risk factors for AKI in diabetic patients include a history of CKD, the presence of heart failure and atherosclerotic disease, hyperglycemic crisis (diabetic ketoacidosis or hyperosmolar hyperglycemic state), the use of antidiabetic medications, and certain interventions such as the use contrast agents^20^. Additionally, the presence of proteinuria is an independent risk factor for AKI in diabetic patients (aOR = 1.158)^12^.

### Pathophysiology

Patients with DKD are at a higher risk of developing AKI because several mechanisms are dysregulated during DKD pathogenesis. The pathophysiology of DKD is complex and multifactorial, involving both metabolic and hemodynamic factors. These factors activate intracellular signaling pathways, increase oxidative stress and hypoxia, dysregulate autophagy, and cause epigenetic changes, leading to kidney inflammation and fibrosis.

Hyperglycemia activates several metabolic pathways, including the polyol pathway, the hexosamine pathway, the protein kinase C (PKC) pathway, and the advanced glycation end-product (AGE) pathway, all leading to the accumulation of reactive oxygen species (ROS)^21^. Excessive ROS are also generated by mitochondrial dysfunction and the upregulation of pro-oxidant enzymes, such as nicotinamide adenine dinucleotide phosphate (NADPH) oxidase, in diabetic kidneys. ROS oxidize important macromolecules, including proteins, lipids, and nucleic acids, ultimately causing significant tissue damage.

Hyperglycemia-associated abnormal glucose metabolism and oxidative stress induce the upregulation of several intracellular signaling pathways that contribute to the pathogenesis of DKD. One such pathway involves mitogen-activated protein kinases (MAPK), whose activation has been shown to induce podocyte apoptosis and extracellular matrix production by mesangial cells. The diabetic milieu also activates Janus kinase-signal transducers and activators of transcription (JAK-STAT) and nuclear factor kappa-B (NF-κB), which are heavily involved in inflammation. NF-κB stimulates adhesion molecules and the expression of proinflammatory cytokines, such as macrophage chemoattractant protein-1 (MCP-1), tumor necrosis factor-α (TNF-α), and interleukin-6 (IL-6), all of which play key roles in the pathogenesis of DKD.

Consequently, metabolic factors such as ROS and AGEs, gut microbiome changes, inflammatory factors like TNF-α, IL-1,IL-6, IL16, IL-18, MCP-1, and matrix metalloproteinase-9 (MMP-9), fibrotic factors including transforming growth factor beta (TGF-β), fibronectin, collagen-1, connective tissue growth factor (CTGF), and hemodynamic factors like endothelin and renin-angiotensin-aldosterone system (RAAS) induce albuminuria and reduce renal function by causing glomerular hypertrophy, mesangial expansion, and tubulointerstitial inflammation^22^. These factors ultimately lead to end-stage kidney disease (ESKD) through a final common pathway involving glomerulosclerosis and tubulointerstitial fibrosis and atrophy.

These findings can be explained by the alterations occurring in the renal microenvironment within the context of DKD, which indicate a maladaptive repair process during AKI. These alterations involve damage to vessels, including glycocalyx changes, pericyte detachment, and endothelial damage, which increase vascular permeability. Ultimately, these changes culminate in proteinuria, chronic inflammation, atherosclerosis, and elevated cardiovascular risk^23^. Moreover, increased levels of ROS and RAAS activation, particularly angiotensin II (Ang II), result in diminished autophagy within podocytes. Therefore, Ang II binds to its angiotensin II type 1 receptor (AT1R) and has a deleterious effect on podocytes by promoting oxidative stress. The Ang II-AT1R signaling promotes the production of hydrogen peroxide (H_2_O_2_) by Nox4, an isoform of NADPH oxidase (Nox) highly expressed in the cortex, representing the main source of ROS generation in the kidney^24^. H_2_O_2_, in turn, acts as a secondary messenger in the cell, and its signaling activates TRPC6 (transient receptor potential cation channel subfamily C member 6), a protein present in the slit diaphragm whose action results in the reorganization of the cytoskeleton both through calcium influx and in a calcium-independent manner. Additionally, the increase in intracellular calcium also results in the phosphorylation and activation of calcineurin, which promotes the translocation of the transcription factor nuclear factor of activated T cells (NFAT) and the increased expression of TRPC6. Thus, there is a persistent positive regulation mediated by Ang II/Nox4/TRPC6 signaling. This, coupled with reduced expression of nephrin, fosters podocyte apoptosis and detachment, consequently disrupting the integrity of the glomerular filtration barrier. These events ultimately lead to proteinuria and tubular stress. In the tubular compartment, heightened glucose-sodium reabsorption in the proximal tubule results in increased Na^+^-K^+^ pump activity, oxygen depletion, endoplasmic reticulum stress, and misfolding of proteins. These changes lead to tubular apoptosis, thereby acutely elevating the risk of hypoxia and ischemia-reperfusion injury, and chronically exacerbating risk of inflammation and fibrosis^23^. More recently, in addition to apoptosis, other programmed cell death pathways have been implicated in the pathogenesis of DKD, including entotic cell death, necroptosis, and pyroptosis^25^.

Therefore, the occurrence of AKI can lead to an increased burden of DKD, as several signaling pathways become dysregulated and may potentially interact, aggravating kidney function and damaging kidney tissue.

### Glycemic Control in the Context of aki and dkd

Variations in glycemic control can consequently amplify the burden of AKI in patients with both DM and DRD.

Increasing evidence indicates the intricate and profound interconnections at multiple levels between various physiological functions including metabolism, immunity, tissue homeostasis, and hematopoiesis^26^. Consequently, the field of immunometabolism shed light on how cells can be programmed by changes in their metabolic environment. Epigenetic modifications, such as changes in DNA (hypo- and –hyper) methylation, are associated with how metabolic signals can induce persistent changes in cell function, despite intensive glucose management^27^.

In the clinical context, hyperglycemia drives changes in bone marrow, endothelial cells, monocytes, macrophages, and adipocytes^26^. In bone marrow, it promotes increased numbers of myeloid progenitor cells and circulating immune cells, whereas endothelial cells, adipocytes, and macrophages are primed for pro-inflammatory gene expression and function, such as NF-κB, intercellular adhesion molecule-1 (ICAM-1), and MCP-1. This persistent low-grade inflammation harms pancreatic beta cells, impairing insulin production and consequently worsening glycemic control, potentially leading to the new-onset hyperglycemia^28^.

Additionally, hyperglycemia hinders neutrophil function by decreasing their rolling, locomotion, migration, phagocytosis, and microbial killing capacity^29^. The reduction in superoxide production by the neutrophil NADPH oxidase may result in a decrease in neutrophil extracellular traps (NETs), which are a crucial antimicrobial mechanism of neutrophilic granulocytes.

Therefore, acute hyperglycemia can significantly alter the innate immune response to infection, potentially exacerbating the poor outcomes observed in hospitalized patients with hyperglycemia. These findings may help explain why patients experiencing new-onset hyperglycemia have an increased risk for ICU admission and mortality^30^.

## Discussion

### Glycemic Control as a Risk Factor for Acute Kidney Injury (aki)

In two large cohorts from the United States and Sweden, it was discovered that levels of glycated hemoglobin (HbA1c) were associated with an elevated risk of AKI. When compared with a baseline HbA1c of 6–6.9%, the hazard ratio (HR) for AKI in patients with HbA1c > 9% ranged from 1.29 to 1.33^31^.

Consequently, the glycemic control of diabetic patients can be affected during hospitalization. For example, knowledge of the risk of AKI in diabetic patients admitted with acute myocardial infarction is of paramount importance as it has therapeutic implications^32^. In the receiver operating characteristic (ROC) curve analyses, the Acute(A)/Chronic(C) glycemic ratio (area under the curve [AUC]: 0.62, p = 0.001) and ΔA−C (AUC: 0.62, p = 0.002) showed higher accuracy than acute glycemia (AUC: 0.57, p = 0.08) for predicting the occurrence of AKI (p = 0.02 and p = 0.01 in comparison with acute glycemia, respectively). There was no difference in AUC between the Acute/Chronic glycemic ratio and ΔA−C (p = 0.53). In the overall population, the cutoff values for the Acute/Chronic glycemic ratio and ΔA−C that maximized sensitivity and specificity for predicting AKI were 1.5 and 106 mg/dL, respectively. Patients with higher values than the cutoffs had a significantly higher incidence of AKI than those with lower values.

In patients with type 1 diabetes mellitus (T1DM) and diabetic ketoacidosis, AKI was diagnosed in 47% of the episodes (stage 1: 46%, stage 2: 43%, and stage 3: 12%) and favored the appearance of microalbuminuria: 1 episode of AKI (HR = 1.33), 2 episodes of AKI (HR = 4.52), 3 episodes of AKI (HR = 8.20), and ≥ 4 episodes of AKI (HR = 9.31)^33^.

In a population of 14,534 individuals with type 2 diabetes mellitus (T2DM), HbA1c values showed a clear association with the risk of developing sepsis compared to values of 6.5–6.9%: <6.1% (aHR [adjusted hazard ratio] = 1.15), 7–7.8% (aHR = 0.93), 7.9–8.7% (aHR = 1.05), 8.8–9.7% (aHR = 1.14), and > 9.7% (aHR = 1.52)^34^. These findings suggest a U-shaped association between HbA1c values and the risk of sepsis, as well as a 4.16-fold increase in mortality among patients who developed sepsis.

### Use of Sodium-Glucose Co-transporters-2 (Sglt2) Inhibitors in the Context of aki

The use of SGLT2 inhibitors has been identified as a protective factor for AKI in several studies (CREDENCE [Canagliflozin and Renal Endpoints in Diabetes with Established Nephropathy Clinical Evaluation], DECLARE-TIMI-58 [Dapagliflozin Effect on Cardiovascular Events], CANVAS [Canagliflozin Cardiovascular Assessment Study], and EMPA-REG) with a relative risk reduction of AKI of 0.75 (95% CI 0.66–0.85)^35^. A more recent meta-analysis involving 13 studies with diabetic patients showed a relative risk reduction of AKI of 0.79 (95% CI 0.72–0.88)^36^.

In diabetic patients with acute myocardial infarction undergoing coronary angioplasty (SGLT2-i AMI Project Registry), the incidence of contrast-induced AKI was 5.4% in those patients using SGLT2 inhibitors (*versus* 13.1%, p = 0.022), with creatinine levels being significantly lower at 72 hours after contrast use^37^. The use of SGLT2 inhibitors was an independent predictor of reduced AKI rate (OR = 0.356, 95% CI 0.134–0.943).

However, for other causes of AKI, such as sepsis, prerenal syndrome, medications, and obstruction, more studies are needed to evaluate the efficacy of SGLT2 inhibitors.

Importantly, euglycemic diabetic ketoacidosis (DKA) is a complication of SGLT2 inhibitor use. Factors related to its occurrence have been described, such as underlying conditions (infection, pulmonary embolism, atrial fibrillation, and AKI itself), dietary modifications (fasting or calorie restriction), and medication changes (use of SGLT2 inhibitors in the perioperative period, including after surgical procedures, and reduction or cessation of insulin)^38^. The primary causes of DKA involve an increase in glucagon levels accompanied by a decrease in insulin levels. This combination leads to heightened protein catabolism and lipolysis, inducing the liver to uptake amino acids and free fatty acids. Consequently, hepatic production and ketoacidosis escalate, alongside increased kidney reabsorption of ketone bodies.

Thus, in these clinical and surgical scenarios, the use of SGLT2 inhibitors should be discontinued. Similarly, other oral antidiabetic medications should be discontinued in diabetic patients, and glycemic control should be managed with insulin.

### Management of Blood Glucose in the Intensive Care Setting

Several large-scale clinical trials have investigated optimal control of acute blood glucose in critically ill patients, including those with sepsis. However, only a few small studies are limited to septic patients, and there is little information specifically for diabetic patients.

Van den Berghe et al.^39^ evaluated patients admitted to surgical ICUs randomly assigned to receive intensive insulin therapy (target blood glucose 80-110 mg/dL) or conventional therapy (target 180-200 mg/dL). Intensive insulin therapy reduced nosocomial sepsis episodes by approximately 46% and the proportion of patients requiring prolonged antibiotic therapy. Specifically, tight glucose control (TGC, i.e. blood glucose levels < 110 mg/dL) was associated with lower rates of morbidity and mortality (with a 43% reduction in relative risk of mortality in the ICU). However, this outcome depended on the benefit of the subgroup of patients who remained in the ICU for more than 5 days and in patients undergoing cardiac surgery (who represented the majority of the study population) and those who received intravenous glucose load previously for nutritional purposes.

In a subsequent study in clinical ICU patients, the same group failed to confirm a mortality benefit in the overall population (40.0% in the conventional-treatment group *versus* 37.3% in the intensive-treatment group, p = 0.33). TGC reduced morbidity, as it prevented newly acquired AKI, accelerated weaning from mechanical ventilation, and accelerated discharge from the ICU and hospital in all patients but reduced mortality only in those who remained in the ICU for at least 3 days (reduction from 52.5% to 43%, p = 0.009)^40^. Additionally, concerns were raised about the high rate of hypoglycemic events (over 6.0 times higher than in the previous study) in this subgroup of patients.

A post-hoc analysis of combined data from the two previous studies further confirmed that TGC had a significantly higher risk of hypoglycemia (which occurred in 11.3% of TGC patients *versus* 1.8% of those with conventional insulin therapy, p < 0.0001)^41^. Intensive insulin therapy, however, decreased mortality rates from 23.6 to 20.4% (p = 0.04) overall, and from 37.9 to 30.1% among long-term patients (p = 0.002). There was no significant difference among short-term patients (8.9 *versus* 10.4%, p = 0.4)^41^.

These combined data thus revealed that TGC significantly reduced morbidity and mortality in both clinical and surgical ICUs (particularly in patients who remained in the ICU for at least 3 days)^39,40,41^. Furthermore, it was reported that all patient subgroups, including those admitted for sepsis, benefited from TGC. However, for diabetic patients, no survival benefit was reported. Rapid normalization of blood glucose levels, rather than hypoglycemic events, was proposed to explain the lack of benefit of TGC in diabetic patients.

Notably, other studies have found no benefits of TGC^42,43,44^, but differences in study design, patient selection, nutritional support, glucose target range, and glucose measurements make comparisons challenging. Indeed, another study specifically involving patients with severe sepsis not only failed to demonstrate a mortality benefit of TGC in diabetic and non-diabetic patients but was prematurely terminated for safety reasons (there was a significantly increased rate of severe hypoglycemic events ≤ 40 mg/dL in the intensive insulin therapy group *versus* conventional therapy group: 17% and 4.1%, respectively, p < 0.001)^44^.

Subsequently, two large-scale clinical trials, including mixed populations of clinical and surgical patients, the Glucontrol study^42^ and the Normoglycemia in Intensive Care Evaluation - Survival Using Glucose Algorithm Regulation (NICE-SUGAR) trial^43^, reported higher rates of hypoglycemia in the TGC group. The first study, prematurely discontinued due to a high rate of unintentional protocol violations, found no differences in TGC mortality, whereas in the NICE-SUGAR study (n = 6,104 patients), intensive glucose control 80-108 mg/dL *versus* > 108 and < 180 mg/dL demonstrated higher mortality (27.5% *versus* 24.9%, respectively, OR 1.14, p = 0.02), regardless of the presence of DM, along with more frequent severe hypoglycemia ≤40 mg/dL (6.8% *versus* 0.5%, p < 0.001). It is worth noting that in the NICE-SUGAR study approximately 20% of patients were diabetic in each arm.

In a meta-analysis study (n = 36 randomized controlled trials with 17,996 patients), Yamada et al.^45^ found no benefit of TGC on mortality, while an increased rate of hypoglycemia was observed in both diabetic and non-diabetic patients under TGC compared to patients under moderate glycemic control (140–180 mg/dL) and very moderate control (180–220 mg/dL). Thus, the U-shaped curve of the relationship between glycemic control and mortality (patients with low and high glucose levels have worse outcomes than those in the normal/moderate range) suggests that a moderately elevated glucose level may represent the ideal target in diabetic patients.

However, further studies are needed to confirm whether these findings are truly due to glucose levels or whether there are confounding variables leading to hypoglycemia and consequently contribute to unfavorable outcomes. Additionally, the relationship between a longer time spent in the 70 to 140 mg/dL glucose range and a lower mortality rate, clearly described in non-diabetic patients, is absent in diabetic patients should also be investigated^46^.

Current guidelines (Surviving Sepsis Campaign) recommend treating hyperglycemia in critically ill patients with a target of 140–180 mg/dL, regardless of previously known DM^47^. The KDIGO-2012 recommendation for glycemic control is insulin treatment aiming for a therapeutic glycemic target of 110–149 mg/dL (evidence 2C)^48^.

The need for specific glycemic control targets in diabetic patients has been postulated, and some studies have suggested that less stringent glycemic control (e.g., targeting blood glucose levels of 180–250 mg/dL) may be beneficial in critically ill patients with pre-morbid chronic hyperglycemia (e.g., HbA1c level >7%)^49,50,51^. However, there are concerns regarding more permissive glucose levels in critically ill diabetic patients, including increased risk of infection, glucosuria, and polyneuropathy. Based on these observations, Egi et al.^49^ proposed adopting a standard blood glucose target for patients with and without DM (≤ 180 mg/dL). More recently, the multicenter, parallel-group, open-label LUCID trial (Liberal Glucose Control in Critically Ill Patients with Preexisting Type 2 Diabetes trial) of 419 adult patients with T2DM admitted to the ICU and randomized into the intervention group (which received intravenous insulin when blood glucose was > 252 mg/dL and titrated to a target range of 180–252 mg/dL) and the comparator group (insulin initiated at a blood glucose >180 mg/dL and titrated to a target range of 108–180 mg/dL)^52^. The primary outcome was the occurrence of hypoglycemia (< 72 mg/dL). Secondary outcomes included glucose metrics and clinical outcomes. By day 28, at least one episode of hypoglycemia occurred in 10 of 210 (5%) patients in the intervention group and in 38 of 209 (18%) patients in the comparator group (incidence rate ratio of 0.21, p < 0.001). Patients in the intervention group had higher blood glucose concentrations (daily mean, minimum, maximum), lower glucose variability, and less relative hypoglycemia (p < 0.001 for all comparisons). By day 90, 62 of 210 (29.5%) patients in the intervention group and 52 of 209 (24.9%) in the comparator group had died (absolute difference of 4.6%, p = 0.29). Thus, a liberal approach to blood glucose targets reduced the incidence of hypoglycemia but did not improve patient-centered outcomes.

Another important aspect is the impact of AKI severity and duration on the risk of hypoglycemia and mortality. The risk of hypoglycemia increases in patients with AKI (OR = 3.6 and aOR = 4.2), so that for each day of AKI duration, there is a 14% increase in the risk of hypoglycaemia^53^. AKI duration is also associated with higher mortality (OR = 1.14, p < 0.001), while the occurrence of hypoglycemia increases mortality risk by 4.4 times.

### Perspectives

The identification of AKI biomarkers in different scenarios has been extensively investigated in the literature to predict acute tubular necrosis, worsening of renal function during hospitalization, and estimated glomerular filtration rate at 6 months, including urinary NGAL (neutrophil gelatinase-associated lipocalin), CXCL9, TNF-α, IL-9, KIM-1 (kidney-injury molecule-1), NAG (N-acetyl-β-D-glucosaminidase), uromodulin, osteopontin, and YKL-40^54^. It would be crucial to conduct an investigation focused on diabetic patients, as the microenvironment in DKD presents peculiarities concerning the dysregulation of various signaling pathways that contribute to disease progression. These pathways may be further exacerbated in the presence of AKI and depending on glycemic control.

Therefore, NAG, which is an early renal damage marker, has been investigated as a candidate for predicting the worsening of DKD in association with other markers (MCP-1, IL-6, and NGAL), independent of proteinuria^55^.

Another investigation should involve AKI biomarkers related to the kidney’s ability to recover after an acute insult, as DKD has a poor renal repair. This investigation would be of paramount importance as failure to recover renal function, regardless of AKI stage, is associated with higher mortality^56^.

Overall, investigations on the molecular subtypes of AKI differ between ischemic and hypoperfusion insults^57^, so identifying a molecular signature of DKD would contribute to the understanding its pathophysiology.

Pharmacologically, it would also be important to advance our understanding of how new medications for slowing the progression of DKD would impact the prevention, attenuation, and recovery of renal function after an acute insult.

## Conclusions

**Figure 1:**
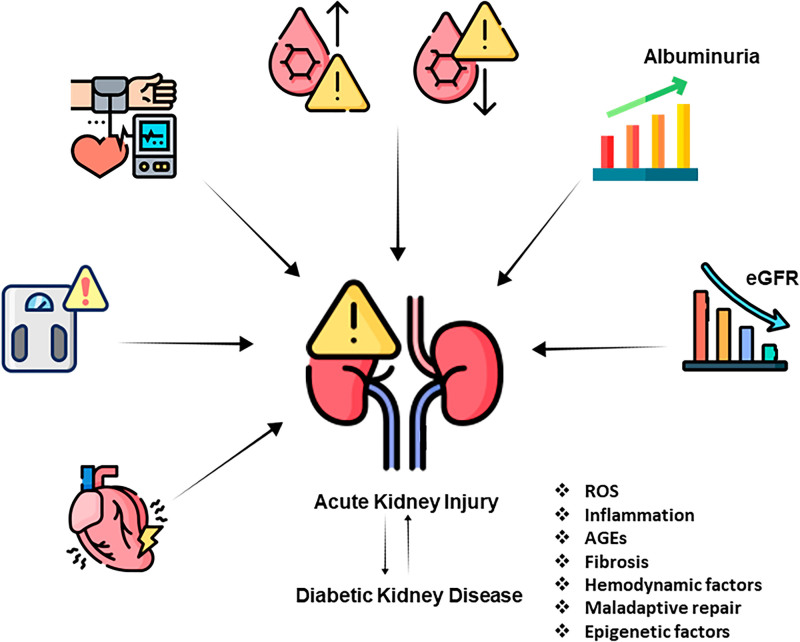
Crosstalk between acute kidney injury (AKI) and diabetic kidney disease (DKD).

Patients with DKD face an elevated risk of developing AKI, which in turn worsens the progression of DKD (Figure 1). Dysregulation in molecular and cellular biological processes within the DKD context contributes to adverse kidney outcomes and patient health following an acute insult.

Glycemic control stands as one of the pillars in mitigating AKI burden on DKD, although the optimal target for glycemic control remains a subject of debate in the literature. For non-diabetic patients admitted to the ICU, glycemic control within the range of 108-180 mg/dL is recommended, as it correlates with reduced mortality and a lower risk of hypoglycemia. Conversely, among diabetic patients admitted to the ICU, glycemic control within the range of 108–180 mg/dL was associated with a higher risk of hypoglycemia compared to control within the range of 180–252 mg/dL, with no discernible differences between the two groups in terms of mortality.

Hence, until further studies comparing glucose targets for diabetic patients are available, the potential benefits of achieving satisfactory glucose levels for diabetic individuals in the ICU should not be outweighed by the risk of hypoglycemia. Conducting randomized controlled studies is essential for assessing the impact of intensive insulin treatment on diabetic patients with DKD and validate AKI biomarkers within this cohort. Such studies will enable the customization of treatment strategies, ultimately enhancing patient outcomes and preserving kidney function.
