# Supplementary material for: Delving into the complexities of the interplay between acute kidney injury and diabetic kidney disease: A focus on glycemic control and outcomes
Source: J Bras Nefrol. 2024 Dec 16;47(1):e20240074. doi: 10.1590/2175-8239-JBN-2024-0074en (PMC11648739; doi:10.1590/2175-8239-JBN-2024-0074en)
Supplement: Translation

# Translation: Investigando as complexidades da interação entre injúria renal aguda e doença renal diabética: Um enfoque no controle glicêmico e desfechos

**DOI:** 10.1590/2175-8239-JBN-2024-0074pt

**Authors:** Érika Bevilaqua Rangel

**Affiliations:** 1 Hospital Israelita Albert Einstein, Instituto Israelita de Ensino e Pesquisa, São Paulo, SP, Brasil.; 2 Universidade Federal de São Paulo, Departamento de Medicina, Divisão de Nefrologia, São Paulo, SP, Brasil.

**Keywords:** Injúria Renal Aguda, Diabetes Mellitus, Doença Renal Diabética, Controle Glicêmico

## Abstract

Pacientes com doença renal diabética (DRD) enfrentam um risco elevado de apresentar injúria renal aguda (IRA), o que agrava a progressão da DRD. Este artigo oferece uma revisão abrangente da literatura e do conhecimento dos principais mecanismos fisiopatológicos relacionados ao dano renal, bem como das implicações biológicas do reparo renal mal adaptativo no contexto da DRD complicada por IRA. Além disso, examinamos detalhadamente os achados de ensaios clínicos que avaliam a eficácia e a segurança do tratamento intensivo com insulina para pacientes hiperglicêmicos em unidades de terapia intensiva, juntamente com os riscos potenciais de hipoglicemia e mortalidade. Ainda, por meio da análise crítica dos resultados de ensaios clínicos, são identificadas oportunidades para abordagens personalizadas baseadas na segurança, a fim de mitigar efeitos colaterais. É imperativo realizar estudos randomizados e controlados para avaliar o impacto do tratamento intensivo com insulina em pacientes diabéticos com DRD e validar os biomarcadores de IRA nessa população de pacientes. Tais estudos ajudarão a adaptar as estratégias de tratamento para melhorar os desfechos dos pacientes e preservar a função renal.

## Introdução

### Epidemiologia

O diabetes mellitus (DM) é um fator de risco significativo para injúria renal aguda (IRA) e apresenta uma prevalência significativamente maior (variando de 19,6% a 49%) em comparação com indivíduos não diabéticos (variando de 9,3% a 33%) em unidades de terapia intensiva (UTIs)^1^. Poucos estudos investigaram a IRA nesse grupo de pacientes. A Tabela 1 fornece um resumo dos estudos relevantes^2,3,4,5,6.7,8,9,10^.

**Tabela 1:** Injúria renal aguda e desfechos em pacientes diabéticos

Estudo	Desenho	Tamanho da amostra (n)	Objetivo	Resultados
Mehta et al.^2^	Análise retrospectiva	449.524	Prevalência de DM em pacientes com IRA após cirurgia cardíaca	Prevalência de DM é maior em pacientes com IRA vs. pacientes sem IRA (49% vs. 33%; p < 0,0001)
Mittalhenkle et al.^3^	Análise prospectiva de caso-controle	5.731	Incidência de IRA na população idosa	DM associado à IRA incidente
Oliveira et al.^4^	Análise prospectiva	980	Prevalência de DM em IRA induzida por aminoglicosídeos	Prevalência de DM é maior em IRA vs. sem IRA (19,6% vs. 9,3%; p = 0,007)
Kheterpal et al.^5^	Análise retrospectiva, baseada em dados (Programa Nacional de Melhoria da Qualidade Cirúrgica do Colégio Americano de Cirurgiões)	75.952	Incidência de IRA após cirurgia geral	Identificação do DM como fator de risco pré-operatório independente
Girman et al.^6^	Análise retrospectiva baseada em dados (General Practice Research Database)	119.966 DMT2 e 1.794.516 indivíduos não diabéticos	IRA em DM vs. não diabéticos	Maior incidência anual de IRA no DM: 198 vs. 27/100.000 indivíduos (HR bruto = 8,0; IC 95%, 7,4–8,7)
Orban et al.^7^	Análise retrospectiva	94	IRA na UTI	Glicemia elevada associada ao aumento da ocorrência de IRA na admissão em UTI
Venot et al.^8^	Análise prospectiva de caso-controle	318 indivíduos diabéticos e 746 não diabéticos	Incidência de IRA em sepse grave/choque séptico	A incidência de IRA não difere. Maior creatinina sérica e frequência de diálise em pacientes diabéticos
Kim et al.^9^	Análise pareada de caso-controle	884	Efeito do DM na IRA após nefrectomia parcial minimamente invasiva	Maior incidência de IRA pós-operatória em DM vs. não DM (30,7% vs. 14,9%; p < 0,001)
Hapca et al.^10^	Análise retrospectiva	9.417 DMT2 e 7.283 indivíduos não diabéticos		Risco de IRA em DM 5 vezes maior do que em controles (48,6% vs. 17,2%; 121,5 vs. 24,6 por 1.000 pessoas-ano)

É importante ressaltar que a maioria dos indivíduos com DM apresenta pelo menos uma doença crônica concomitante, e até 40% têm ao menos três ou mais condições, incluindo doença renal diabética (DRD), hipertensão, insuficiência cardíaca, doença vascular periférica, obesidade, entre outras^11^. Dessa forma, esses fatores contribuem para um alto risco de IRA.

As principais causas de IRA em pacientes diabéticos são sepse (61%), síndrome cardiorrenal (11%), síndrome pré-renal (9,9%), medicamentos (9%), obstrução (6,6%) e uso de contraste (4%)^12^.

A IRA induzida por medicamentos é um desafio significativo na prática clínica. A nefrotoxicidade associada a medicamentos é responsável por 18–27% de todos os casos de IRA em hospitais americanos^13^. No estudo DIRECT (*Drug-Induced Renal Injury Consortium*), os medicamentos nefrotóxicos mais comuns causadores de IRA foram identificados como vancomicina (48,7%), anti-inflamatórios não esteroides (AINEs; 18,2%) e piperacilina/tazobactam (17,8%)^14^. Além da hiperglicemia, um modelo multivariado identificou idade, capacidade vascular, infecções, piúria, tendência da creatinina sérica e meios de contraste como preditores significativos de IRA induzida por medicamentos, demonstrando um bom desempenho com uma AUC-ROC (Área sob a Curva de Característica Operacional do Receptor ou Area Under the Receiver Operating Characteristic Curve) de 0,86.

Os mecanismos fisiopatológicos da IRA induzida por medicamentos variam de acordo com o medicamento utilizado^13^. Os inibidores da enzima conversora de angiotensina (inibidores da ECA) e os bloqueadores dos receptores da angiotensina (BRAs) são os principais contribuintes, responsáveis por 35% dos casos de IRA, principalmente devido ao seu uso generalizado em pacientes diabéticos. O risco é ainda mais elevado em pacientes com insuficiência cardíaca congestiva, depleção de volume, aqueles em uso de diuréticos ou AINEs e em pacientes com estenose bilateral da artéria renal. Os aminoglicosídeos (como a gentamicina) e os AINEs são responsáveis por 16% dos casos, seguidos por estatinas (10%), rifampicina (6%) e ifosfamida (3%). Os AINEs reduzem a taxa de filtração glomerular e o fluxo sanguíneo renal ao suprimir a produção de prostaglandina.

A IRA associada a diuréticos é caracterizada pela vacuolização de células epiteliais tubulares e é mais frequentemente observada em pacientes com maiores taxas de comorbidades (DM, doença cardiovascular, doença renal crônica e hipertensão) em comparação com aqueles com IRA não associada a diuréticos^15^. No grupo de IRA induzida por diuréticos, 27,5% dos casos foram atribuídos exclusivamente a diuréticos, enquanto 29,8% resultaram da combinação de diuréticos com outros medicamentos. Um estudo retrospectivo revelou que pacientes diabéticos em uso de hidroclorotiazida (HCT) frequentemente apresentaram eventos renais significativos, com um declínio da TFGe superior a 30% em cerca de 20% dos indivíduos^16^.

É importante ressaltar que a hipocalemia induzida por diuréticos também pode agravar o controle glicêmico ou promover a hiperglicemia, uma vez que a hipocalemia prejudica a liberação de insulina dependente de potássio em resposta à sobrecarga de glicose^17^.

A evolução dos pacientes diabéticos que desenvolvem IRA indica que cerca de 10% apresentam um declínio na taxa de filtração glomerular estimada (TFGe) e 47% apresentam um declínio de mais de 50% na TFG, especialmente se a causa da IRA for sepse^12^.

O risco de IRA é diretamente proporcional à TFG (em mL/min/1,73 m^2^): 40–50 (*odds ratio* ajustado [aOR] 1,95), 30–44 (aOR 6,54), 15–29 (aOR 28,50) e < 15 (aOR 40,07)^18^. Se outras comorbidades estiverem presentes, como insuficiência cardíaca e hipertensão arterial sistêmica, o risco de IRA aumenta (OR = 3,37 e 1,94, respectivamente)^19^.

É importante notar que as taxas de IRA em pacientes diabéticos com doença renal crônica (DRC) são duas vezes mais elevadas do que no grupo não diabético. Após o início da DRC, as taxas de IRA foram de 384,8 por 1.000 pessoas-ano para pacientes diabéticos, em comparação com 180,0 por 1.000 pessoas-ano para não diabéticos^10^. Antes do início da DRC, as taxas eram de 109,3 por 1.000 pessoas-ano para pacientes diabéticos *vs.* 47,4 por 1.000 pessoas-ano para não diabéticos.

Portanto, os fatores de risco para IRA em pacientes diabéticos incluem histórico de DRC, presença de insuficiência cardíaca e doença aterosclerótica, crise hiperglicêmica (cetoacidose diabética ou estado hiperglicêmico hiperosmolar), uso de medicamentos antidiabéticos e determinadas intervenções, como o uso de agentes de contraste^20^. Além disso, a presença de proteinúria é um fator de risco independente para IRA em pacientes diabéticos (aOR = 1,158)^12^.

### Fisiopatologia

Os pacientes com DRD apresentam maior risco de desenvolver IRA em razão da desregulação de diversos mecanismos durante a patogênese da DRD. A fisiopatologia da DRD é complexa e multifatorial, envolvendo fatores metabólicos e hemodinâmicos. Esses fatores ativam vias de sinalização intracelular, aumentam o estresse oxidativo e a hipóxia, desregulam a autofagia e causam alterações epigenéticas, resultando em inflamação e fibrose renal.

A hiperglicemia ativa várias vias metabólicas, incluindo a via do poliol, a via da hexosamina, a via da proteína quinase C (PKC) e a via do produto final de glicação avançada (AGE), todas levando ao acúmulo de espécies reativas do oxigênio (ERO)^21^. O excesso de EROs também é gerado pela disfunção mitocondrial e pela superexpressão de enzimas pró-oxidantes, como a nicotinamida adenina dinucleotídeo fosfato (NADPH) oxidase, nos rins diabéticos. As EROs oxidam macromoléculas importantes, incluindo proteínas, lipídios e ácidos nucleicos, causando, por fim, danos teciduais significativos.

O metabolismo anormal da glicose associado à hiperglicemia e o estresse oxidativo induzem a regulação positiva de diversas vias de sinalização intracelular que contribuem na patogênese da DRD. Uma dessas vias envolve proteínas quinases ativadas por mitógenos (MAPK), cuja ativação tem demonstrado induzir a apoptose de podócitos e a produção de matriz extracelular por células mesangiais. O ambiente diabético também ativa a via de sinalização Janus quinase/transdutores de sinal e ativadores de transcrição (JAK-STAT) e o fator nuclear kappa-B (NF-κB), que estão fortemente envolvidos na inflamação. O NF-κB estimula moléculas de adesão e a expressão de citocinas pró-inflamatórias, como a proteína quimioatraente de macrófagos-1 (MCP-1), o fator de necrose tumoral-α (TNF-α) e a interleucina-6 (IL-6), que desempenham papéis fundamentais na patogênese da DRD.

Consequentemente, fatores metabólicos como ERO e AGEs, alterações no microbioma intestinal, fatores inflamatórios, como TNF-α, IL-1, IL-6, IL-16, IL-18, MCP-1 e metaloproteinase de matriz-9 (MMP-9), fatores fibróticos, incluindo o fator de crescimento transformador beta (TGF-β), fibronectina, colágeno-1, fator de crescimento do tecido conjuntivo (CTGF) e fatores hemodinâmicos, como endotelina e o sistema renina-angiotensina-aldosterona (SRAA), induzem a albuminúria e reduzem a função renal ao causar hipertrofia glomerular, expansão mesangial e inflamação tubulointersticial^22^. Esses fatores acabam levando à doença renal em estágio terminal (DRET) por meio de uma via final comum que envolve glomeruloesclerose e fibrose e atrofia tubulointersticial.

Esses achados podem ser explicados pelas alterações que ocorrem no microambiente renal no contexto da DRD, indicando um processo de reparo mal adaptativo durante a IRA. Essas alterações envolvem danos aos vasos, incluindo alterações no glicocálix, destacamento de pericitos e danos endoteliais, que aumentam a permeabilidade vascular. Em última instância, essas alterações culminam em proteinúria, inflamação crônica, aterosclerose e risco cardiovascular elevado^23^. Além disso, níveis aumentados de EROs e a ativação do SRAA, especialmente a angiotensina II (Ang II), resultam em diminuição da autofagia nos podócitos. Portanto, a Ang II se liga ao seu receptor tipo 1 da angiotensina II (AT1R) e exerce um efeito deletério nos podócitos ao promover o estresse oxidativo. A sinalização Ang II-AT1R promove a produção de peróxido de hidrogênio (H2O2) pela Nox4, uma isoforma de NADPH oxidase (Nox) altamente expressa no córtex, representando a principal fonte de geração de ERO no rim^24^. O H2O2, por sua vez, atua como um mensageiro secundário na célula, e sua sinalização ativa o TRPC6 (canal catiônico potencial de receptor transitório, subfamília C, membro 6), uma proteína presente no diafragma de fenda, cuja ação resulta na reorganização do citoesqueleto tanto por meio do influxo de cálcio quanto de forma independente do cálcio. Além disso, o aumento do cálcio intracelular também resulta na fosforilação e ativação da calcineurina, que promove a translocação do fator de transcrição NFAT (fator nuclear de células T ativadas) e o aumento da expressão de TRPC6. Assim, há uma regulação positiva persistente mediada pela sinalização Ang II/Nox4/TRPC6. Isso, juntamente com a expressão reduzida de nefrina, promove a apoptose e o desprendimento de podócitos, comprometendo, consequentemente, a integridade da barreira de filtração glomerular. Esses eventos acabam levando à proteinúria e ao estresse tubular. No compartimento tubular, o aumento da reabsorção de glicose e sódio no túbulo proximal resulta em maior atividade da bomba de Na^+^-K^+^, depleção de oxigênio, estresse de retículo endoplasmático e dobramento incorreto de proteínas. Essas alterações levam à apoptose tubular, consequentemente elevando de forma aguda o risco de hipóxia e lesão de isquemia-reperfusão, e exacerbando de forma crônica o risco de inflamação e fibrose^23^. Mais recentemente, além da apoptose, outras vias de morte celular programada têm sido implicadas na patogênese da DRD, incluindo morte celular entótica, necroptose e piroptose^25^.

Portanto, a ocorrência de IRA pode levar a uma carga aumentada de DRD, uma vez que diversas vias de sinalização se tornam desreguladas e podem interagir potencialmente, agravando a função renal e danificando o tecido renal.

### Controle Glicêmico no Contexto de ira e drd

Variações no controle glicêmico podem, consequentemente, amplificar a carga de IRA em pacientes com DM e DRD.

Evidências crescentes indicam as interconexões intrincadas e profundas em múltiplos níveis entre diversas funções fisiológicas, incluindo metabolismo, imunidade, homeostase tecidual e hematopoiese^26^. Consequentemente, o campo do imunometabolismo lançou luz sobre como as células podem ser programadas por alterações em seu ambiente metabólico. Modificações epigenéticas, como alterações na (hipo- e hiper) metilação do DNA, estão associadas à forma como os sinais metabólicos podem induzir alterações persistentes na função celular, apesar do manejo intensivo da glicose^27^.

No contexto clínico, a hiperglicemia promove alterações na medula óssea, células endoteliais, monócitos, macrófagos e adipócitos^26^. Na medula óssea, promove um aumento no número de células progenitoras mieloides e células imunes circulantes, enquanto células endoteliais, adipócitos e macrófagos são preparados para a expressão e função de genes pró-inflamatórios, como NF-κB, molécula de adesão intercelular-1 (ICAM-1) e MCP-1. Essa inflamação crônica de baixo grau prejudica as células beta pancreáticas, comprometendo a produção de insulina e, consequentemente, piorando o controle glicêmico, podendo levar à hiperglicemia de início recente^28^.

Além disso, a hiperglicemia prejudica a função dos neutrófilos ao reduzir sua capacidade de rolagem, locomoção, migração, fagocitose e morte microbiana^29^. A redução na produção de superóxido pela NADPH oxidase de neutrófilos pode resultar em uma diminuição das armadilhas extracelulares dos neutrófilos (NETs), que são um mecanismo antimicrobiano crucial dos granulócitos neutrofílicos.

Portanto, a hiperglicemia aguda pode alterar significativamente a resposta imune inata à infecção, potencialmente exacerbando os desfechos desfavoráveis observados em pacientes hospitalizados com hiperglicemia. Esses achados podem ajudar a explicar por que pacientes com hiperglicemia de início recente apresentam um aumento no risco de admissão em UTI e de mortalidade^30^.

## Discussão

### Controle Glicêmico como Fator de Risco Para Injúria Renal Aguda (ira)

Em duas grandes coortes dos Estados Unidos e da Suécia, descobriu-se que níveis de hemoglobina glicada (HbA1c) estavam associados a um risco elevado de IRA. Quando comparada a uma HbA1c basal de 6–6,9%, a razão de riscos (HR) para IRA em pacientes com HbA1c > 9% variou de 1,29 a 1,33^31^.

Consequentemente, o controle glicêmico de pacientes diabéticos pode ser afetado durante a hospitalização. Por exemplo, ter conhecimento do risco de IRA em pacientes diabéticos internados com infarto agudo do miocárdio é de suma importância, uma vez que possui implicações terapêuticas^32^. Nas análises da curva ROC (*Receiver Operating Characteristic*), a razão glicêmica Aguda(A)/Crônica(C) (área sob a curva [AUC]: 0,62; p = 0,001) e o ΔA−C (AUC: 0,62; p = 0,002) mostraram maior precisão do que a glicemia aguda (AUC: 0,57; p = 0,08) para prever a ocorrência de IRA (p = 0,02 e p = 0,01 em comparação com a glicemia aguda, respectivamente). Não houve diferença na AUC entre a razão glicêmica Aguda/Crônica e o ΔA−C (p = 0,53). Na população geral, os valores de corte para a razão glicêmica Aguda/Crônica e ΔA−C que maximizaram a sensibilidade e especificidade para a predição de IRA foram 1,5 e 106 mg/dL, respectivamente. Pacientes com valores superiores aos de corte apresentaram uma incidência significativamente maior de IRA do que aqueles com valores inferiores.

Em pacientes com diabetes mellitus tipo 1 (DMT1) e cetoacidose diabética, a IRA foi diagnosticada em 47% dos episódios (estágio 1: 46%, estágio 2: 43% e estágio 3: 12%) e favoreceu o aparecimento de microalbuminúria: 1 episódio de IRA (HR = 1,33), 2 episódios de IRA (HR = 4,52), 3 episódios de IRA (HR = 8,20) e ≥ 4 episódios de IRA (HR=9,31)^33^.

Em uma população de 14.534 indivíduos com diabetes mellitus tipo 2 (DMT2), os valores de HbA1c mostraram uma clara associação com o risco de desenvolver sepse em comparação com valores de 6,5–6,9%: < 6,1% (aHR [razão de riscos ajustada] = 1,15), 7–7,8% (aHR = 0,93), 7,9–8,7% (aHR = 1,05), 8,8–9,7% (aHR = 1,14) e >9,7% (aHR = 1,52)^34^. Esses achados sugerem uma associação em U entre os valores de HbA1c e o risco de sepse, bem como um aumento de 4,16 vezes na mortalidade entre os pacientes que desenvolveram sepse.

### Uso de Inibidores do Cotransportador de Sódio-glicose-2 (sglt2) no Contexto da ira

O uso de inibidores de SGLT2 foi identificado como um fator de proteção para IRA em diversos estudos (CREDENCE [*Canagliflozin and Renal Endpoints in Diabetes with Established Nephropathy Clinical Evaluation*], DECLARE-TIMI-58 [*Dapagliflozin Effect on Cardiovascular Events*], CANVAS [*Canagliflozin Cardiovascular Assessment Study*] e EMPA-REG) com uma redução do risco relativo de IRA de 0,75 (IC 95% 0,66–0,85)^35^. Uma meta-análise mais recente envolvendo 13 estudos com pacientes diabéticos mostrou uma redução do risco relativo de IRA de 0,79 (IC 95% 0,72–0,88)^36^.

Em pacientes diabéticos com infarto agudo do miocárdio submetidos a angioplastia coronária (Registro do Projeto SGLT2-i AMI), a incidência de IRA induzida por contraste foi de 5,4% entre os pacientes em uso de inibidores de SGLT2 (*versus* 13,1%; p = 0,022), com níveis de creatinina significativamente mais baixos em 72 horas após o uso do contraste^37^. O uso de inibidores de SGLT2 foi um preditor independente da redução na taxa de IRA (OR = 0,356; IC 95% 0,134–0,943).

No entanto, para outras causas de IRA, como sepse, síndrome pré-renal, medicamentos e obstrução, são necessários mais estudos para avaliar a eficácia dos inibidores de SGLT2.

É importante ressaltar que a cetoacidose diabética (CAD) euglicêmica é uma complicação do uso de inibidores de SGLT2. Foram descritos fatores relacionados à sua ocorrência, como condições subjacentes (infecção, embolia pulmonar, fibrilação atrial e a própria IRA), modificações na dieta (jejum ou restrição calórica) e alterações medicamentosas (uso de inibidores de SGLT2 no período perioperatório, inclusive após procedimentos cirúrgicos, e redução ou interrupção da insulina)^38^. As principais causas da CAD envolvem um aumento nos níveis de glucagon acompanhado de uma diminuição nos níveis de insulina. Essa combinação leva ao aumento do catabolismo proteico e da lipólise, induzindo o fígado a absorver aminoácidos e ácidos graxos livres. Consequentemente, a produção hepática e a cetoacidose aumentam, acompanhadas do aumento da reabsorção renal de corpos cetônicos.

Assim, nesses cenários clínicos e cirúrgicos, o uso de inibidores de SGLT2 deve ser descontinuado. Da mesma forma, outros medicamentos antidiabéticos orais devem ser descontinuados em pacientes diabéticos, e o controle glicêmico deve ser manejado com insulina.

### Manejo da Glicemia no Ambiente de Terapia Intensiva

Diversos ensaios clínicos de larga escala investigaram o controle ideal da glicemia aguda em pacientes críticos, incluindo aqueles com sepse. No entanto, somente alguns pequenos estudos se limitam a pacientes sépticos, e há poucas informações específicas para pacientes diabéticos.

Van den Berghe et al.^39^ avaliaram pacientes admitidos em UTIs cirúrgicas, distribuídos aleatoriamente para receber terapia intensiva com insulina (meta de glicemia de 80–110 mg/dL) ou terapia convencional (meta de 180–200 mg/dL). A terapia intensiva com insulina reduziu os episódios de sepse nosocomial em aproximadamente 46%, bem como a proporção de pacientes que necessitaram de terapia antibiótica prolongada. Especificamente, o controle glicêmico rigoroso (TGC, por sua sigla em inglês), ou seja, níveis de glicose no sangue < 110 mg/dL, foi associado a taxas mais baixas de morbidade e mortalidade (com uma redução de 43% no risco relativo de mortalidade na UTI). No entanto, esse desfecho dependeu do benefício do subgrupo de pacientes que permaneceram na UTI por mais de 5 dias e em pacientes submetidos à cirurgia cardíaca (que representaram a maioria da população do estudo) e daqueles que receberam previamente uma carga de glicose intravenosa para fins nutricionais.

Em um estudo subsequente com pacientes de UTI clínica, o mesmo grupo não conseguiu confirmar um benefício de mortalidade na população geral (40,0% no grupo de tratamento convencional *versus* 37,3% no grupo de tratamento intensivo, p = 0,33). O TGC reduziu a morbidade, uma vez que preveniu um episódio primário de IRA, acelerou o desmame da ventilação mecânica e agilizou a alta da UTI e do hospital em todos os pacientes. Porém, reduziu a mortalidade somente naqueles que permaneceram na UTI por pelo menos 3 dias (redução de 52,5% para 43%; p = 0,009)^40^. Além disso, surgiram preocupações sobre a taxa elevada de eventos hipoglicêmicos (mais de 6 vezes maior do que no estudo anterior) nesse subgrupo de pacientes.

Uma análise post-hoc de dados combinados dos dois estudos anteriores confirmou ainda que o TGC apresentou um risco significativamente maior de hipoglicemia (ocorrendo em 11,3% dos pacientes com TGC *versus* 1,8% daqueles com terapia convencional com insulina, p < 0,0001)^41^. A terapia intensiva com insulina, no entanto, reduziu as taxas de mortalidade de 23,6% para 20,4% (p = 0,04) no geral, e de 37,9% para 30,1% entre os pacientes de longo prazo (p = 0,002). Não houve diferença significativa entre os pacientes de curto prazo (8,9 *versus* 10,4%; p = 0,4)^41^.

Esses dados combinados revelaram, portanto, que o TGC reduziu significativamente a morbidade e a mortalidade tanto em UTIs clínicas quanto cirúrgicas (particularmente em pacientes que permaneceram na UTI por pelo menos 3 dias)^39,40,41^. Além disso, foi relatado que todos os subgrupos de pacientes, incluindo aqueles internados por sepse, beneficiaram-se do TGC. No entanto, para pacientes diabéticos, não foi relatado nenhum benefício de sobrevida. A rápida normalização dos níveis de glicose no sangue, em vez de eventos hipoglicêmicos, foi proposta para explicar a falta de benefício do TGC em pacientes diabéticos.

Notavelmente, outros estudos não encontraram benefícios do TGC^42,43,44^, mas diferenças no desenho do estudo, seleção de pacientes, suporte nutricional, intervalo alvo de glicose e medições de glicose tornam as comparações desafiadoras. De fato, outro estudo envolvendo especificamente pacientes com sepse grave não apenas falhou em demonstrar um benefício de mortalidade do TGC em pacientes diabéticos e não diabéticos, como também foi encerrado prematuramente por razões de segurança (houve um aumento significativo na taxa de eventos hipoglicêmicos graves ≤ 40 mg/dL no grupo de terapia intensiva com insulina *versus* o grupo de terapia convencional: 17% e 4,1%, respectivamente, p < 0,001)^44^.

Posteriormente, dois ensaios clínicos de larga escala, incluindo populações mistas de pacientes clínicos e cirúrgicos, o estudo Glucontrol^42^ e o ensaio NICE-SUGAR (*Normoglycemia in Intensive Care Evaluation - Survival Using Glucose Algorithm Regulation*)^43^, relataram taxas mais elevadas de hipoglicemia no grupo TGC. O primeiro estudo, interrompido precocemente devido a uma alta taxa de violações não intencionais de protocolo, não encontrou diferenças na mortalidade do TGC. Por outo lado, no estudo NICE-SUGAR (n = 6.104 pacientes), o controle glicêmico intensivo (80–108 mg/dL) *versus* > 108 e < 180 mg/dL demonstrou maior mortalidade (27,5% *versus* 24,9%, respectivamente, OR 1,14; p = 0,02), independentemente da presença de DM, juntamente com maior frequência de hipoglicemia grave ≤ 40 mg/dL (6,8% *versus* 0,5%). Vale ressaltar que, no estudo NICE-SUGAR, aproximadamente 20% dos pacientes em cada braço eram diabéticos.

Em um estudo de meta-análise (n = 36 ensaios clínicos randomizados com 17.996 pacientes), Yamada et al.^45^ não encontraram benefício do TGC na mortalidade, enquanto uma taxa aumentada de hipoglicemia foi observada tanto em pacientes diabéticos como não diabéticos sob TGC, em comparação com pacientes sob controle glicêmico moderado (140–180 mg/dL) e controle muito moderado (180–220 mg/dL). Assim, a curva em U da relação entre controle glicêmico e mortalidade (pacientes com níveis baixos e elevados de glicose apresentam piores desfechos em comparação com aqueles na faixa normal/moderada) sugere que um nível de glicose moderadamente elevado pode representar a meta ideal em pacientes diabéticos.

No entanto, são necessários mais estudos para confirmar se esses achados são realmente decorrentes dos níveis de glicose ou se existem variáveis de confusão que levam à hipoglicemia e, consequentemente, contribuem para desfechos desfavoráveis. Além disso, a relação entre um tempo mais longo gasto no intervalo glicêmico de 70 a 140 mg/dL e uma menor taxa de mortalidade, claramente descrita em pacientes não diabéticos, está ausente em pacientes diabéticos e também deve ser investigada^46^.

As diretrizes atuais (Campanha de Sobrevivência à Sepse) recomendam o tratamento da hiperglicemia em pacientes críticos com uma meta de 140–180 mg/dL, independentemente de DM previamente conhecido^47^. A recomendação do KDIGO-2012 para o controle glicêmico é o tratamento com insulina visando a uma meta glicêmica terapêutica de 110-149 mg/dL (evidência 2C)^48^.

A necessidade de metas específicas de controle glicêmico em pacientes diabéticos tem sido postulada, e alguns estudos sugerem que um controle glicêmico menos rigoroso (por exemplo, visando níveis de glicose no sangue de 180–250 mg/dL) pode ser benéfico em pacientes críticos com hiperglicemia crônica pré-mórbida (por exemplo, nível de HbA1c > 7%)^49,50,51^. No entanto, existem preocupações quanto a níveis de glicose mais permissivos em pacientes diabéticos críticos, incluindo o risco aumentado de infecção, glicosúria e polineuropatia. Com base nessas observações, Egi et al.^49^ propuseram a adoção de uma meta padrão de glicemia para pacientes com e sem DM (≤ 180 mg/dL). Mais recentemente, o ensaio multicêntrico, de grupo paralelo e aberto LUCID (*Liberal Glucose Control in Critically Illents with Preeististed Type 2 Diabetes*), com 419 pacientes adultos com DMT2 admitidos na UTI, foi randomizado em dois grupos: o grupo intervenção (que recebeu insulina intravenosa quando a glicemia era > 252 mg/dL e titulada para um intervalo alvo de 180–252 mg/dL) e o grupo comparador (insulina iniciada com uma glicemia > 180 mg/dL e titulada para um intervalo alvo de 108–180 mg/dL)^52^. O desfecho primário foi a ocorrência de hipoglicemia (< 72 mg/dL). Os desfechos secundários incluíram métricas de glicose e desfechos clínicos. Até o 28° dia, ocorreu pelo menos um episódio de hipoglicemia em 10 dos 210 (5%) pacientes no grupo intervenção e em 38 dos 209 (18%) pacientes no grupo comparador (razão de taxa de incidência de 0,21; p < 0,001). Os pacientes no grupo intervenção apresentaram concentrações mais elevadas de glicose no sangue (média, mínimo e máximo diários), menor variabilidade glicêmica e menos hipoglicemia relativa (p < 0,001 para todas as comparações). Até o dia 90, 62 dos 210 (29,5%) pacientes no grupo intervenção e 52 dos 209 (24,9%) no grupo comparador haviam ido a óbito (diferença absoluta de 4,6%, p = 0,29). Assim, uma abordagem liberal às metas de glicemia reduziu a incidência de hipoglicemia, mas não melhorou os desfechos centrados no paciente.

Outro aspecto importante é o impacto da gravidade e da duração da IRA no risco de hipoglicemia e mortalidade. O risco de hipoglicemia aumenta em pacientes com IRA (OR = 3,6 e aOR = 4,2), de modo que, para cada dia de duração da IRA, há um aumento de 14% no risco de hipoglicemia^53^. A duração da IRA também está associada a uma maior mortalidade (OR = 1,14; p < 0,001), enquanto a ocorrência de hipoglicemia aumenta o risco de mortalidade em 4,4 vezes.

### Perspectivas

A identificação de biomarcadores de IRA em diferentes cenários tem sido amplamente investigada na literatura para prever necrose tubular aguda, piora da função renal durante a hospitalização e taxa de filtração glomerular estimada em 6 meses, incluindo NGAL (lipocalina associada à gelatinase de neutrófilos) urinária, CXCL9, TNF-α, IL-9, KIM-1 (molécula de lesão renal-1), NAG (N-acetil-β-D-glicosaminidase), uromodulina, osteopontina e YKL-40^54^. Seria crucial realizar uma investigação focada em pacientes diabéticos, uma vez que o microambiente na DRD apresenta peculiaridades relacionadas à desregulação de diversas vias de sinalização que contribuem para a progressão da doença. Essas vias podem ser ainda mais exacerbadas na presença de IRA e dependendo do controle glicêmico.

Portanto, a NAG, que é um marcador de dano renal precoce, tem sido investigada como candidata para predizer o agravamento da DRD em associação com outros marcadores (MCP-1, IL-6 e NGAL), independentemente da proteinúria^55^.

Outra investigação deve envolver biomarcadores de IRA relacionados à capacidade do rim de se recuperar após um insulto agudo, já que a DRD apresenta um reparo renal deficiente. Essa investigação seria de suma importância, uma vez que a falha na recuperação da função renal, independentemente do estágio da IRA, está associada a uma maior mortalidade^56^.

De modo geral, as investigações sobre os subtipos moleculares de IRA diferem entre insultos isquêmicos e de hipoperfusão^57^, portanto, a identificação de uma assinatura molecular da DRD contribuiria para a compreensão de sua fisiopatologia.

Do ponto de vista farmacológico, também seria importante avançar em nossa compreensão de como novos medicamentos para retardar a progressão da DRD teriam impacto na prevenção, atenuação e recuperação da função renal após um insulto agudo.

## Conclusões

Os pacientes com DRD enfrentam um risco elevado de desenvolver IRA, o que, por sua vez, agrava a progressão da DRD (Figura 1). A desregulação dos processos biológicos moleculares e celulares no contexto da DRD contribui para desfechos renais adversos e para a saúde do paciente após um insulto agudo.

**Figura 1:** *Crosstalk* entre injúria renal aguda (IRA) e doença renal diabética (DRD).

O controle glicêmico permanece como um dos pilares na mitigação da carga de IRA na DRD, embora a meta ideal para o controle glicêmico continue sendo tema de debate na literatura. Para pacientes não diabéticos internados na UTI, recomenda-se o controle glicêmico dentro do intervalo de 108–180 mg/dL, uma vez que esse controle está relacionado à redução da mortalidade e ao menor risco de hipoglicemia. Por outro lado, entre pacientes diabéticos internados na UTI, o controle glicêmico no intervalo de 108–180 mg/dL foi associado a um risco maior de hipoglicemia em comparação com o controle na faixa de 180–252 mg/dL, sem diferenças perceptíveis entre os dois grupos em termos de mortalidade.

Dessa forma, até que mais estudos comparando as metas glicêmicas para pacientes diabéticos estejam disponíveis, os potenciais benefícios de atingir níveis glicêmicos satisfatórios para indivíduos diabéticos na UTI não devem ser superados pelo risco de hipoglicemia. A realização de estudos randomizados e controlados é essencial para avaliar o impacto do tratamento intensivo com insulina em pacientes diabéticos com DRD e validar biomarcadores de IRA nessa coorte. Esses estudos permitirão a personalização das estratégias de tratamento, melhorando, em última análise, os desfechos dos pacientes e preservando a função renal.
